# Prospective Case–Control Study of Determinants for African Swine Fever Introduction in Commercial Pig Farms in Poland, Romania, and Lithuania

**DOI:** 10.1155/tbed/5419764

**Published:** 2025-05-22

**Authors:** S. Dhollander, E. Cattaneo, J. Cortiñas Abrahantes, A. E. Boklund, A. Szczotka-Bochniarz, A. D. Mihalca, A. Papanikolaou, L. Mur, O. M. Balmoș, M. Frant, A. Gal-Cisoń, M. Kwasnik, W. Rozek, A. Malakauskas, M. Masiulis, J. Turčinavičienė, A. Rusinà, R. Aminalragia-Giamini, T. Chesnoiu, K. Jazdzewski, J. Rola, F. Barbuceanu, J. A. Stegeman

**Affiliations:** ^1^Assess/Enable Department, European Food Safety Authority, Parma, Italy; ^2^Department of Veterinary and Animal Sciences, University of Copenhagen, Copenhagen, Denmark; ^3^Department of Cattle and Sheep Diseases, National Veterinary Research Institute, Pulawy, Poland; ^4^Department of Parasitology and Parasitic Diseases, University of Agricultural Sciences and Veterinary Medicine, Cluj-Napoca, Romania; ^5^Department of Swine Diseases, National Veterinary Research Institute, Pulawy, Poland; ^6^Department of Virology, National Veterinary Research Institute, Pulawy, Poland; ^7^Veterinary Academy, Lithuanian University of Health Sciences, Kaunas, Lithuania; ^8^Department of Zoology, Institute of Biosciences, Life Sciences Centre of Vilnius University, Vilnius, Lithuania; ^9^Department of Animal Health and Welfare, National Sanitary Veterinary and Food Safety Authority, Bucharest, Romania; ^10^Chief Veterinary Office, General Veterinary Inspectorate, Warsaw, Poland; ^11^Pathology Department, Institute for Diagnosis and Animal Health, Bucharest, Romania; ^12^Department of Clinical Sciences 2, University of Agronomical Sciences and Veterinary Medicine of Bucharest, Bucharest, Romania; ^13^Department of Farm Animal Health, Utrecht University, Utrecht, Netherlands

## Abstract

To investigate potential husbandry-related risk factors for African swine fever (ASF) incursion on commercial pig farms in Lithuania, Poland, and Romania, a prospective, matched case–control design was carried out from August 2021 to September 2023. For each participating commercial pig farm where an ASF outbreak occurred, two control farms were randomly selected and matched by herd size and county. On both case and control farms, questionnaires related to farm management and biosecurity measures were carried out, and stable flies (*Stomoxys calcitrans*) and biting midges (Culicoides spp.) were collected to identify a possible association between the number of ASF virus (ASFV)- or ASFV DNA-positive vectors and presence of ASF on the farms. After testing for potential multicollinearity, a conditional logistic regression model was performed on one complete and three imputed datasets. To evaluate the best-fit model, the Akaike information criterion (AIC) method was used. This study generated more knowledge on risk and protective factors for ASF introduction on commercial pig farms related to (1) farm location (risk: closer distance to ASF outbreaks); (2) the wild boar (WB)–pig interface (risk: attractive crops for WB cultivated near the farms); (3) biosecurity (protective: carcasses collection by the rendering company without entering the holding and closed containers for carcass disposal, risk: sharing machinery with other farms or organizing unusual events on the farm); (4) insect-mediated mechanic transmission (protective: placement of insect screens on all doors and windows and risk: the number of biting midges collected on the farm). Manure from other holdings applied on the soil near the farm was in two of four models found significant and could be related to possible mechanical transmission by stable flies or to an increased infection pressure. Some of the identified husbandry-related risks and protective factors can have a direct practical value for the farmers.

## 1. Introduction

Since the introduction of African swine fever (ASF) into Georgia in 2007 from its originally endemic area in Africa, the global spread of ASF has caused a devastating economic impact on pig production and pervasive ecological consequences due to the impact on wild boar (WB) populations as well as on the conservation of various endangered wild suids. Between 2022 and 2024, the presence of the virus was reported to WOAH in all five world regions, and the reported outbreaks affected more than 489,000 pigs and more than 18,700 WBs, causing more than 1,390,000 killed or dead animals [1].

Whereas in the domestic pig (DP) sector, limited between farm spread has been observed in most countries, a self-sustaining infection cycle in WB in Eastern and Central Europe has been established despite all the implemented control measures [2].

In the DP sector, human activities are considered the main driver of the disease spread. However, the exact route of virus introduction into farms is rarely elucidated [3]. A systematic literature review carried out in 2021 demonstrated that breaches in farm biosecurity and increased farm activity were linked to an increased risk of ASF virus (ASFV) introduction on pig farms [4]. Most risk factor studies, found in the review, originate from the studies that used readily available observations on environmental, ecological, or demographical parameters to make inferences on their possible correlation with ASF occurrence on pig farms or WB. Few studies were identified where quantitative data on specific farm practices had been collected to estimate their association with the introduction of the virus. The identification of specific farm-management-related risk factors is of crucial importance, as these risk factors could possibly be mitigated by farmers or risk managers, if made aware of them. Risk factors related to the surrounding landcover or location of the farm are primarily of academic interest, as there is little that can be done about them. Boklund et al. [5] conducted a case–control study in 2019 on Romanian backyard and commercial farms to identify risk factors associated with ASFV introduction. The proximity to outbreaks in domestic farms was a risk factor for both commercial and backyard farms. Furthermore, in backyard farms, factors such as herd size, WB abundance near the farm, the number of domestic outbreaks within 2 km around farms, proximity to WB ASF cases, and visits by professionals working on farms were statistically significant risk factors. Additionally, growing crops around the farm, which could potentially attract WB, and feeding forage from ASF-affected areas to the pigs were identified as risk factors for ASF incursion in backyard farms. The limited number of outbreaks in commercial pig farms resulted in reduced power to identify specific farm management-related risk factors, should they be present.

The observed seasonality of ASF outbreaks in DPs, with an increased incidence during summer [6], raises hypotheses of risk factors linked to farming practices, such as crop harvesting and summer feeding practices, or changes in WB behavior during summer, leading to more frequent WB–DP interactions [3].

Contradictorily, the persistence of ASFV increases with decreasing temperatures. A recent study by Blome et al. [7] analyzed the survival of ASFV in feed and bedding materials under different ambient conditions. The detection of infectious virus was limited to temperatures below 10°C in all feed and bedding materials studied, while the viral genome could be detected—for example, on potatoes, for up to 274 days at 10°C and up to 90 days at 20°C. However, an expert knowledge elicitation conducted in 2021 by EFSA concluded that the lowest risk from all assessed matrices for introducing infections on pig farms would be from bedding/enrichment materials (sawdust, straw, and wooden toys) and forage [4].

The summer peak in ASF incidence on pig farms could, however, also be linked to biological or mechanical transmission of the virus by vectors, which might explain its introduction on farms where strict biosecurity measures were implemented. Both potential biological transmission [8, 9] and mechanical transmission [10, 11] of the Georgia 2007/1 strain of ASFV has been the subject of several studies since its introduction in Europe. However, the role of the biological vector, *Ornithodoros erraticus* sensu lato (s.l.), in the spread of ASFV in the currently affected areas in north-eastern Europe is unlikely, as this species of soft tick has not yet been observed in these regions. Additionally, *O. erraticus* s.l. is less efficient in transmitting Georgia 2007/01 to pigs compared to *Ornithodoros moubata*, the vector prevalent in endemic African countries [11].

Blome et al. [7] isolated ASFV from *Stomoxys calcitrans* flies kept in an incubator at 20°C up to 42 h after feeding on viremic blood but only up to 3 h from *Aedes albopictus* kept under the same conditions. Several studies demonstrated the ASFV genome to be present in *S. calcitrans* flies collected in the field [12, 13] and in Culicoides spp. on farms with ASF outbreaks [14], suggesting that these insects could potentially play a role in mechanical transmission of ASFV. The mechanical transmission of ASFV by *S. calcitrans* has been demonstrated by Mellor et al. [15] in laboratory conditions. However, the contribution of insect-mediated mechanical transmission of the virus in the field, relative to other transmission routes, remains to be clarified.

This study is a follow-up of the case–control study conducted by Boklund et al. [5] mentioned above, aiming to identify specific husbandry-related risk factors for the introduction of ASFV on commercial pig farms. To increase the power of the study to identify such factors in commercial pig farms, outbreaks in Lithuania and Poland, in addition to Romania, were included. Additionally, vectors were collected from both outbreak and control farms, with the objective of identifying a possible association between the number of ASFV and ASFV DNA-positive vectors and the presence of the disease on the farms.

## 2. Materials and Methods

### 2.1. Study Design

To investigate potential risk factors for ASF incursion, a prospective matched case–control design was used. This study focused on identifying the risk factors for the introduction of ASFV on commercial farms, defined as farms where pigs are bred for commercial purposes. For each outbreak farm, two matched control farms were randomly selected, matched by herd size (e.g., 30–200 pigs; 201–1000 pigs; >1000 pigs) and by Nomenclature of Territorial Units for Statistics (NUTS) 3 region. If a control farm within the same NUTS3 region could not be found, a control farm from the same size category was chosen within the country. The objective was to visit case farms within 2 days of ASF confirmation and control farms within 2 weeks following the outbreak confirmation at the matched outbreak farm.

**Figure 1:**
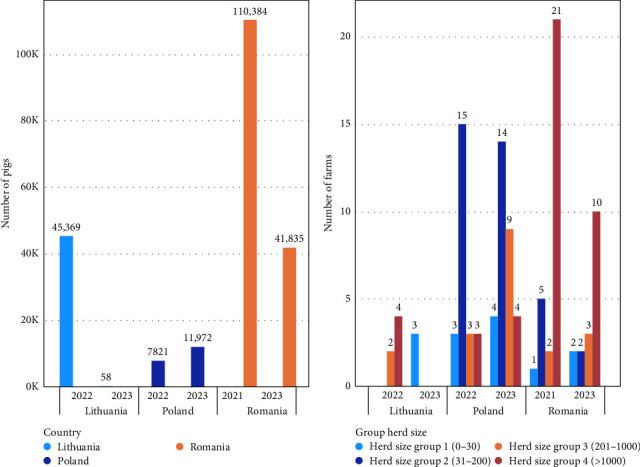
Total number of pigs, farms, and herd size category per country per year included in the African swine fever (ASF) case–control study. (A) Total number of pigs per country per year. (B) Number of farms per year, by country, and herd size category.

Between August 4, 2021, and August 23, 2022, all commercial pig farms in Romania, Lithuania, and Poland with more than 30 pigs where ASF was diagnosed and that were willing to collaborate were included as case farms in the study. To achieve a study power of at least 0.9 for detecting an odds ratio as low as 3, it was initially planned to obtain a minimum of 90 case farms and 180 control farms in total. However, due to the limited number of outbreaks in larger commercial farms, only 26 case farms and 46 control farms met the requirements and agreed to participate within 2 years. As a result, for the third year, the inclusion criteria were expanded to include commercial farms with more than 10 pigs in ASF outbreaks, and an additional herd size class was added (10–30 pigs; 31–200 pigs; 201–1000 pigs and >1000 pigs) (Figure 1) in the study and matched with control farms. Consequently, between May 18, 2023, and September 27, 2023, another 22 case farms and 42 control farms were included in the study.

All eligible outbreak farms were contacted by an official veterinarian and invited to participate in the study. The official veterinarians visited each farm and completed a questionnaire regarding potential risk factors related to management and biosecurity measures. The questionnaire comprised 42 questions covering the following topics: number and age groups of pigs (six questions), slaughter practices, pig outdoor access and other animal species kept in the holding (five questions), presence of WB in the vicinity of the farm (six questions), feed and water management (eight questions), indirect contacts, such as vehicles and visitors (three questions), bedding, manure and fencing (four questions), contacts with other farms during the high-risk period (six questions) and observations of ticks, mosquitoes and midges (four questions). The complete questionnaire is available in the Supporting Information (S1). The high-risk period was defined as the time when ASF introduction might have occurred undetected on the farm and was consistent with the study by Boklund et al. [5], where the high-risk period was assumed 6 weeks before the outbreak confirmation.

For both outbreak and control farms, the distances to other outbreaks in the surrounding area during the high-risk period were calculated. The geographic coordinates of the farms in the surrounding areas were obtained from the Animal Disease Notification System (ADNS). With these calculated distances, three covariates were defined for use in the final conditional logistic regression model: (i) the distance to the nearest WB ASF case, (ii) the distance to the nearest ASF outbreak in DPs, and (iii) the total numbers of cases in WB or outbreaks in DPs within 1, 5, 10, and 15 km. Additionally, two covariates related to the farm surroundings were calculated using the 2018 raster version of CORINE Land Cover data: (iv) the percentage of forest cover in each NUTS 3 unit, where the outbreak and control farms were located, and (v) the presence of water bodies within 1 km of the outbreak or control farms recorded as a binomial variable (yes/no). Both the percentage of forest cover and the presence of water bodies were extracted from the 2018 raster version of CORINE Land Cover data.

### 2.2. Collection of Vectors

On both case and control farms, *S. calcitrans* flies were collected using sticky traps [16], with two traps placed inside and two outside each pig shed. Additionally, biting midges (Culicoides spp.) were collected using MiniCDC traps equipped with UV light [17]. For midges, two traps were deployed—one inside and one outside the pig sheds—on both control and case farms. Vector surveillance only took place in the warmer months, that is, from May onward until and including October, depending on the year.

Identification of the arthropods was performed morphologically using a binocular zoom microscope. After identifying the vector species, the *S. calcitrans* and Culicoides spp. samples from each farm were pooled. Stable flies were pooled into groups of up to four individuals, and biting midges were pooled into groups of up to 10 specimens, following the method of Şevik and Oz [18]. The pools were then tested for ASFV using real-time PCR and ASFV isolation, according to the protocols outlined in the ASF EURL SOPs. The sampling protocol for each group is detailed in the manuscript submitted by Dhollander et al. [19].

### 2.3. Data Analysis

#### 2.3.1. Univariate Analysis

To avoid multicollinearity, potential risk factors were initially assessed using variance inflation factor (VIF) analysis, and only those with VIF values below 5 were retained for the model-building process [20, 21]. Subsequently, where possible and depending on the number of observations, each of the remaining variables was described using descriptive statistics in the univariate analyses.

The questionnaire and vector survey generated 42 covariates (Table A1). To simplify the analysis, and due to a smaller sample size than initially planned, categorical variables were transformed into binary variables. In addition, some covariates were omitted from the analysis because they had uniform responses (e.g., rodent control was everywhere implemented, swill feeding was nowhere implemented, and WBs were almost never observed around the farms or feed stores). To address ambiguous responses such as “I don't know” or blank answers in the questionnaire, four different datasets were created. The first dataset, termed the “complete dataset,” included only variables without ambiguous answers, implying removal of four variables due to “I don't know” or blank responses. The second dataset, the “positive imputed dataset,” converted all “I don't know” answers to “yes.” The third dataset, (the “negative imputed dataset”), converted “I don't know” answers to “no.” The fourth dataset used a random forest model to impute “I don't know” answers. The imputation of missing values used the missForest package [22]. Here, missing values are initially replaced by the mean (for continuous variables) or the most frequent class (for categorical variables). The algorithm then uses random forests to iteratively predict and impute missing values. The process is done sequentially based on the number of missing values in each variable, with the missing data being treated as the test set and the observed data as the training set. The imputation process is repeated until the difference between successive imputations stabilizes, ensuring that the best possible estimates are obtained. Stability is assessed by comparing the change in imputed values between iterations, using the normalized root mean squared error for continuous variables or the proportion of falsely classified entries for categorical variables.

**Figure 2:**
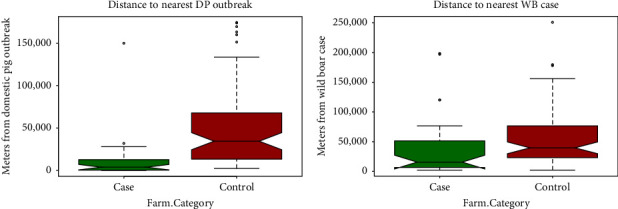
Box plots of the distance from case and control farms to the nearest outbreak in domestic pigs (A) and wild boar (B).

Figure 2 in Figure 5 illustrates the four variables for which “I do not know” responses were recorded: (1) contact with outbreak farms during the high-risk period; (2) application of manure from other holdings near the farms during the high-risk period; (3) outdoor activities performed by staff (such as hunting, mushroom picking, and hiking); and (4) unusual events that occurred during the high-risk period.

A Fisher's exact test was used for the categorical variables, while the Wilcoxon signed-rank test was applied to continuous variables. The results are presented in Table 1. Due to the small sample size, variables with a *p*-value below 0.2 were considered significant in the univariate analysis (indicated in bold in Table 1).

#### 2.3.2. Multivariate Analysis

All data were analyzed using conditional logistic regression models with disease status (outbreak or control farm) as the outcome variable and the covariates described above as explanatory variables. Control farms were matched to outbreak farms based on size and county.

Initially, all possible explanatory variables were included in the multivariate models. Stepwise backward elimination was performed manually, where the variable with the highest *p*-value was sequentially excluded from the model. Once the final model was identified, Akaike information criterion (AIC)-based model selection was used to evaluate it. Additionally, meaningful interactions were tested using a comparison of the AIC obtained from the models, including the interaction and the model excluding them. The relevant interactions that were tested were the interactions between the number of pigs, sightings of WBs around farms, WB abundance, and pig density. The test performed revealed that the interactions considered were not relevant.

## 3. Results

**Figure 3:**
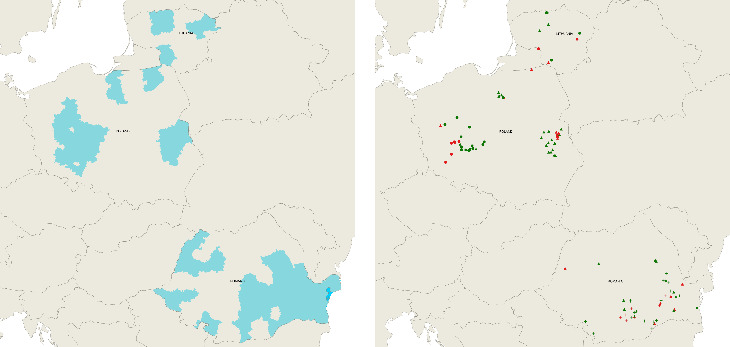
Location of the farms in Lithuania, Poland, and Romania that were included in the study: counties (A) and point locations in the different years (+ = 2021, circle = 2022, triangle = 2023) and categories (red = case farms, green = control farms) (B).

In total, between August 4 and September 27, 2023, 136 farms were included in the analyses: 55 farms in Poland (19 case farms and 36 control farms), 72 in Romania (26 case farms and 46 control farms), and nine in Lithuania (three case and six control farms). This was about half of the 270 case and control farms that were originally planned to be included in the study. The main reason for this reduced number of included farms was the low number of outbreaks in commercial pig farms that met the inclusion criteria during the study period.  Figure 3A shows the different counties where these farms were situated, and Figure 3B shows the locations of the farms over the different years when the study was performed.

From the 136 farms that were taking part in the study, only 110 farms (37 case farms and 73 control farms) were included in the statistical analysis because in the remaining 26 farms, either the questionnaire or the vector survey was not carried out, or there was only one matching control farm visited instead of two, so the farms needed to be removed from the study due to incomplete datasets.

### 3.1. Descriptive Statistics and Univariate Analysis

At first, the VIF analysis was performed to test for potential multicollinearity. The results are listed in Table A2, with 32 variates that remained for the complete dataset, 35 covariates that remained for the “positive” dataset, 32 covariates remaining for the “negative” dataset, and 33 covariates remaining for the imputed database with random forest model. On the remaining variables for each dataset, a univariate analysis was performed, considering all relevant variables with a *p*-value below 0.2. Table 1 summarizes the results of the univariate analysis. As expected, the results that were obtained for the variables that were kept in the best-fit model for the complete dataset were the same as for the imputed datasets.

#### 3.1.1. Observations Related to the WB–DP Interface

From the 110 farms that remained for the statistical analysis, 64 had attractive crops cultivated in the vicinity of the farm (within 100 m to the holding), and 106 farms had a fence around the farm. On 14 of the farms, the farmers or workers reported to be involved in outdoor activities. Both “farmers or workers involved in outdoor activities” and “attractive crops in the vicinity of the farms” were significantly associated with the case in the univariate analysis.

#### 3.1.2. Observations Related to Feed and Water Provided to the Pigs

Sixty of the 110 farms in the analysis provided only tap water to the pigs, while the other 50 farms also provided water from other sources, such as groundwater from a well, rainwater stored on the farm, or river/lake water. Only 11 farms provided hay or fresh grass to the pigs. None of the variables related to feed and water provided to the pigs were significant in the univariate analysis.

#### 3.1.3. Observation Related to Events in the High-Risk Period

Eleven farmers reported unusual events (e.g., break-in security routine, change of staff, new construction building, or social event) taking place on their farm, and 27 reported the introduction of pigs into the herd in the high-risk period (6 weeks before confirmation in the case farm or similar period in control farms). Nine farmers had contact with farmers from outbreak farms during the HRP. “Unusual events that took place during the high-risk period,” “contacts with outbreak farms,” and “number of professional visitors on the farm (e.g., veterinarians) during the high-risk period” were significant in the univariate analysis.

#### 3.1.4. Observations Related to Biosecurity Measures Implemented on the Farm

From all the 110 farms that entered the univariate analysis, 66 farmers provided bedding to the pigs, 13 farms performed on-farm slaughter of pigs, on 54 farms other animals were kept, 95 farms had facilities to disinfect vehicles before entering the farm, 57 farms had a closed storage place for carcasses; on 60 farms the feed could be delivered without entering the premises; 50 farms had an all-in-all-out farming system, and 11 farms shared machinery with other farms. Only the variable “shared machinery” during the high-risk period was significant in the univariate analysis.

#### 3.1.5. Observations Related to Location of the Farms

On 26 of the 110 farms, manure from other holdings was spread out on the field in the close vicinity of the farms. The median distance to the nearest DP outbreak in case farms was 3.8 km, while this was 34.5 km in control farms. The median distance to the nearest ASF-positive WB for case farms was 15.6 km, while this was 39.6 km for control farms.

The median number of outbreaks in a radius of 15 km was 2 and 0 outbreaks for case and control farms, respectively. There was no difference in the median WB abundance, suitable WB habitat, and forest coverage, being 3, 0.2%, and 0%, respectively. The 75th percentiles were 0.2, 0.4, and 0 for the case farms and 5.5, 0.3, and 0 for the control farms, respectively. The distance to the nearest DP outbreak (Figure 2A), the distance to the nearest WB outbreak (Figure 2B), the number of DP outbreaks within 15 km, and the percentage of water in the 2x2 square km in which the farm is located were significant variables.

#### 3.1.6. Observations Related to Potential Transmission of ASFV by Arthropod Vectors

Of the 110 farms included in the univariate analysis, 62 farms had insect nets installed on all the windows, and 76 used insecticides in the sheds. The median of the total number of biting midges caught on each case and control farms was 30 and 18 midges, respectively, ranging from zero to 370 midges per farm. Of those, seven pools were PCR positive on the case farms and one pool on the control farms. The median of the total number of *S. calcitrans* flies caught on each case and control farms was zero and two flies, respectively, ranging from zero to 123 flies caught per farm, of which two pools were testing PCR positive on the case farms, and no pools were positive on the control farms.

Significant variables in the univariate analysis were the use of insecticide, the proportion of positive midges and positive *S. calcitrans flies*, and the total number of *S. calcitrans flies* caught on the farms.

## 4. Conditional Logistic Regression Analysis

Following the univariate analysis, a conditional logistic regression model was performed initially on all variables with a VIF value smaller than 5 for both complete and imputed datasets. Progressively, all the variables with the highest *p* value were removed from the model. A cutoff value of 0.1 was set to determine which variables were significant. To evaluate the best fit model for the conditional logistic regression, the AIC method was used.

### 4.1. Complete Dataset

The output from the AIC function showed that the best-fit model includes four variables with an AIC value of 42.13 (Table 2).

Three of these variables (attractive crops, carcasses collection, and minimum distance from DP outbreaks) reported a *p*-value below 0.05 and hence were considered significant risk factors. It should be noted that both the minimum distance to the nearest DP outbreak and the carcass storage facilities are protective factors, meaning that larger distances to the nearest DP outbreak are associated with smaller odds of having an outbreak on the farm. Also, farms that have carcass collection by rendering companies that do not have to enter the farm premises are less likely to have outbreaks.

The odds ratios demonstrated, for instance, that farms that have attractive crops cultivated in the vicinity are eight times more likely to get ASF outbreaks.

### 4.2. Imputed Dataset With Random Forest Model

The output of the AIC function showed that the best model includes four variables with an AIC of 45.41 (Table 3).

All these variables reported a *p*-value below 0.05; hence, they were considered significant risk factors. The odds ratios demonstrated, for instance, that farms that use bedding material are about eight times more likely to get ASF outbreaks.

### 4.3. Positive Dataset

The output from the AIC function showed that the best model includes six variables with an AIC value of 35.83 (Table 4).

All these variables reported a *p*-value below 0.05; hence, they were considered significant risk factors. The only variable that had a contra-intuitive result in the positive dataset was the average number of nonprofessional visitors. This was caused by one outlier, that is, a big social event in one of the control farms. The odd ratios demonstrated, for instance, that farms that have attractive crops for WB cultivated in the vicinity are about 10 times more likely to get ASF outbreaks.

### 4.4. Negative Dataset

The output from the AIC function showed that the best model included six parameters with an AIC value of 54.67 for the “Negative” dataset (Table 5).

Of the six variables included, four of them reported a *p*-value below 0.05; hence, they were considered significant (attractive crops, unusual events, carcasses storage, and number of *S. calcitrans* flies). The number of *S. calcitrans* flies had a contra intuitive result in the multivariate analysis of the negative dataset. This is caused again by one outlier, a control farm that did not have insect nets and had a very large number of *S. calcitrans* flies present on that control farm. The odd ratios demonstrated, for instance, that farms that have attractive crops cultivated in the vicinity are about nine times more likely to get ASF outbreaks.

### 4.5. Model Output Comparison

**Figure 4:**
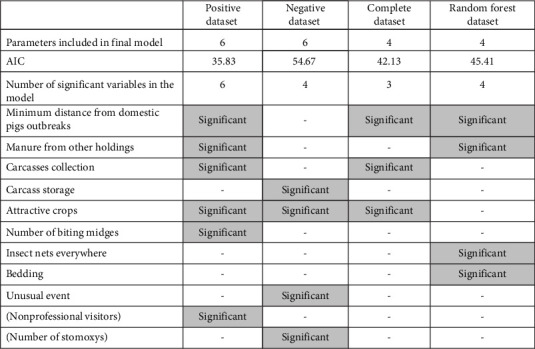
Summary of best-fit model outputs of different datasets. “—“: Variable not included in the best-fit model. Significant: *p*  ≤ 0.5.

**Figure 5:**
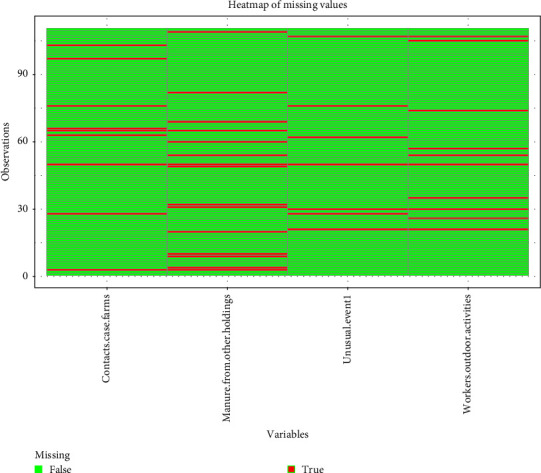
Heat map for ambiguous responses in four questions of questionnaire. Red: response = I do not know; Green: response = completed variable. Heatmap of ambiguous responses to the questionnaire in 110 pig farms located in Romania, Lithuania, and Poland. The heatmap displays the frequency of ambiguous answers (in red) on the four questions: “Was there contact with outbreak farms during the high-risk period?”; “Was manure applied from other holdings in the vicinity of the farm?”; “Did unusual events take place during the high-risk periods?” and “Are workers involved in outdoor activities?” The other questions did not have ambiguous answers and were not displayed. All these ambiguous answers were provided on control farms.

Figure 4 summarizes the significant variables that were obtained by the conditional logistic regression analysis of the four datasets. It is important to note that the results for each of the best-fit model outputs of different datasets cannot be compared using the AIC. As they are derived from different data, the results of the likelihood function on which the AIC is based, do not necessarily correspond between the datasets. However, it is important to highlight that the likelihood that variables that were significant in three of the best-fit models represent a risk factor for the presence of ASF in the commercial farms is higher than those that were significant in only one of the models.

## 5. Discussion

Two covariates consistently emerged as significant risk factors in three of the best-fitting versions of the four models: “Minimum distance (m) to the nearest domestic pig outbreak” and the presence of “attractive crops for WB cultivated near the farms.” Two additional covariates were significant in two of the best-fit models”: Manure from other holdings applied on the soil near the farm,” which was identified, and ”carcasses collection by the rendering company without entering the holding,” which was considered a protective factor. Three covariates appeared as significant risk factors in only one of the models (“number of biting midges on the farm,” “bedding applied on the farm,” and an “unusual event taking place during the high-risk period”). Additionally, two factors were identified as protective factors in only one of the models (“closed carcasses storage” and “insect nets applied on all the windows and doors”). The two remaining significant protective factors identified by only one of the models (“the number of nonprofessional visitors” and “the number of *S. calcitrans* flies”) lack biological plausibility and are likely due to outliers. These outliers were caused by a big party organized on one of the control farms and the presence of a high number of stable flies on another control farm that did not use insect nets that had a very high number of stable flies present on the farm. These outliers were kept in the data for the sake of transparency.

The “Minimum distance to nearest domestic pig outbreak” was consistent with the findings of Boklund et al. [5]. “Growing attractive crops around the farm” also emerged as a significant risk factor in all four models, highlighting the importance of the interface between WB and the DP sector. This suggests the possible spread of the virus by WB roaming near farms despite 96% of the farms in the analysis being fenced. The study did not investigate the type or integrity of the fences. Surprisingly, the distance to the nearest outbreak in WB appeared not significant in the multivariate analysis. Beyond potential limitations in statistical power, this may also reflect undetected viral circulation in WB populations. Additionally, the observed distribution of infected WB may be influenced by where carcasses are more likely to be found—such as near walking paths or hunting areas—while, in reality, the animals may spend more time near crop fields during the summer. As a result, the location of detected cases may not accurately represent the true spatial distribution of infections.

The ASFV DNA-positive midges observed in a control farm located 131 km from the nearest reported outbreaks also points at such unnoticed spread, as the typical range of biting midges is usually not more than 1 km [23]. Cross-contamination of samples was considered unlikely due to the use of different traps and collection teams for case and control farms.

Regarding possible vector transmission, two statistically significant variables may be explained by mechanical transmission through vectors. The placement of insect screens on all doors and windows was found to be a significant protective factor, while the number of midges caught on farms was identified as a significant risk factor. Also, the use of manure from other farms could be explained either increase infection pressure or attract stable flies.

In Lithuania, ASFV DNA has been previously identified in stable flies on outbreak farms [13] and on a high-biosecurity pig farm that experienced an outbreak 2 years earlier [12]. In Romania, ASF DNA was detected on 13 out of 15 outbreak farms sampled in 2020, specifically in 51 of 81 pools of two to three stable flies each [14]. Additionally, Balmos et al. [14] detected ASFV DNA in 50 out of 119 Culicoides spp. pools taken from 11 out of 20 outbreak farms sampled during the same study in 2020.

In the present study, ASFV DNA was detected in both biting midges and stable flies on outbreak farms and control farms; however, no ASFV was isolated. To our knowledge, this is the first prospective case–control study to demonstrate a significant difference in risk factors associated with potential mechanical transmission by arthropod vectors. It is reasonable to argue that the higher probability to detecting ASFV DNA on outbreak farms, compared to control farms, is due to the presence of infected blood and secreta on the outbreak farms, where arthropods may land and feed. This, however, does not proof their involvement in mechanic transmission.

Nonetheless, the detection of ASFV DNA-positive midges and *S. calcitrans* flies on control farms suggests these insects could serve as vehicles for spreading the virus to noninfected farms. The timing and temperature during the sampling of the insects are crucial, as demonstrated by Blome et al. [7], who showed that 2 days with temperatures exceeding 20°C are sufficient to inactivate the virus. A notable outcome of this study is the significant difference in the use of insect nets between case and control farms, suggesting insect nets can help prevent the introduction of infected arthropods. Therefore, the study confirms that installing intact insect screens on all windows and doors of commercial pig farms is a good investment, besides maintaining strict biosecurity measures. Breaches in biosecurity measures, such as sharing machinery with other farms or organizing unusual events on the farm, were identified as risk factors. On the other hand, carcass collection in a dedicated place outside the farm and keeping carcasses in special storage containers proved to be protective factors.

The ambiguous responses from farm managers to certain questions suggest a possible lack of knowledge about their staff's activities or the extent to which biosecurity measures are followed on the farms. This was evident from the frequent answers “I do not know” answers to questions related to outdoor activities of staff (*n* = 10), unusual events organized on the farms (*n* = 7), contact with outbreak farms during the high-risk period (*n* = 7) and the use of manure from other farms applied on the fields nearby their farm (*n* = 15). To minimize recall bias, interviews were conducted as soon as possible after ASF was detected on the outbreak farm. On case farms, interviews were completed within 2 days after ASF confirmation, while 62 control farms (85%) were visited within 2 weeks of the matching outbreak farm's confirmation. Another six farms (8%) were visited between 2 and 3 weeks after, and five farms (7%) were visited after 3 weeks with a maximum delay of 29 days. Therefore, recall bias is unlikely to account for the farmers' uncertainty in answering some of the questions. Whilst all ambiguous answers were given on control farms, the time that had elapsed between the end of the high-risk period and the day of the questionnaire was maximum a month.

To address the few ambiguous answers, four datasets were created: one omitting the ambiguous variables, one imputing them as positive, one as negative, and one generating them randomly. The conditional logistic regression analysis of the four datasets resulted in several significant variables that were all biologically plausible, except for two variables, which could be explained by some notable outliers. However, a better understanding of the farmers on the meaning of the questions could have avoided the need to create alternative datasets and would have increased the power of the study. This is particularly important given that the number of outbreaks in large commercial pig farms in Lithuania, Romania, and Poland during the study period was smaller than anticipated during the design of the prospective case–control study.

It should be noted that the lower-than-expected number of outbreaks in commercial pig farms during the study period necessitated some adjustments in the statistical analysis. Certain categorical variables with several answer categories were grouped into binary categories, and some variables were excluded to mitigate multicollinearity, which may have led to a loss of specific details regarding production systems. Despite these necessary simplifications, our analysis still yielded significant findings that contribute novel insights into risk factors associated with ASFV outbreaks in commercial pig farms. In conclusion, after 3 years of study, and thanks to the cross-border collaboration of farmers, veterinary services, and researchers of Lithuania, Romania, and Poland, more knowledge has been generated into risk factors for the introduction of ASF on commercial pig farms. The results align with previous studies on backyard farms Boklund et al. [5], highlighting consistently significant factors such as proximity to the nearest outbreak, the WB–DP interface, breaches in biosecurity during the high-risk periods, and the potential transmission by mechanical vectors.

Finally, we recommend that future studies should consider tailoring questionnaires to specific commercial production systems to better capture specific farm management practices. In particular, a more comprehensive assessment of cleaning and disinfection protocols would be beneficial. Evaluating the implementation of structured biosecurity measures, such as the separation of clean and dirty areas and the frequency and methods of cleaning, could provide a deeper understanding of their role in ASFV prevention. While our study did include questions on vehicle contacts and disinfection wheels, these factors were not found to be significant. However, future research could further explore these aspects in greater detail, especially in relation to specific production types and biosecurity practices.

It is important to note that increasing the number of questions in the questionnaire would require a larger sample size to maintain statistical power in a case–control study. However, obtaining such a large sample size is challenging, as it would necessitate a sufficient number of outbreaks within specific commercial production systems. Therefore, while expanding the scope of questionnaires could enhance the accuracy of risk assessments, it is crucial to balance this with the practical limitations of outbreak data availability.

## Figures and Tables

**Table 1:** Results of the univariate analysis.

	Complete dataset					Imputed dataset		
	Covariate*⁣*^*∗*^	Count of 37 case farms	Count of 73 control farms	Count of all 110 farms	*p*-Value complete dataset^a^	*p*-Value imputed (RF)^b^	*p*-Value positive dataset^c^	*p*-Value negative dataset^d^
Wild boar–domestic pig interface	Fenced holding	36	70	106	1	1	1	1
	Outdoor activities	8	6	14	**NA**	**0.03623**	**0.006469**	**0.06771**
	Attractive crops	27	37	64	**0.04001**	**0.04001**	**0.04001**	**0.04001**

Feed and water	Tap only	22	38	60	0.5448	—	0.5448	—
	Grass hay	4	7	11	1	1	1	1

Events in the high-risk period	Unusual event	9	2	11	**NA**	**0.0008285**	**1.756e–07**	**0.0008285**
	Pigs introduced	8	19	27	0.6484	0.6484	0.6484	0.6484
	Contact with outbreak farms	7	2	9	**NA**	**0.006461**	**0.0003047**	**0.006461**
	Professional visitors	305	279	584 (sum of visitors)	**0.1567**	**0.1567**	**0.1567**	**0.1567**
	Nonprofessional visitors	35	76	111 (sum of visitors)	0.3116	0.3116	0.3116	0.3116

Biosecurity measures implemented in the farm	Bedding	25	41	66	0.3051	0.3051	0.3051	0.3051
	On-farm slaughter	6	7	13	0.3551	0.3551	0.3551	0.3551
	Other animals	20	34	54	0.546	—	0.546	—
	Disinfection	32	63	95	1	1	1	1
	Carcass storage	19	38	57	1	1	1	1
	Workers food	7	12	19	0.7923	0.7923	0.7923	0.7923
	Carcasses collection	30	63	93	0.5781	0.5781	0.5781	0.5781
	Feed delivery	19	41	60	0.6878	0.6878	0.6878	0.6878
	All in all out	16	34	50	0.8401	0.8401	0.8401	0.8401
	Shared machinery	6	5	11	**0.1769**	**0.1769**	**0.1769**	**0.1769**

Location and size of the farm	Total pigs	77,051 (range: 11−27,069)	140,388 (range: 1–16,817)	217,439 (range: 0–34,234)	0.8125	0.8125	0.8125	0.8125
	Manure from other holdings	11	15	26	NA	0.02659	**0.03762**	0.3438
	Near DP	3.8 km (75th% = 12.6 km)	34.5 km (75th% = 67.9 km)	21.7 km	**2.34e–09**	**2.34e–09**	**2.34e–09**	**2.34e–09**
	Near WB	15.6 Km (75th% = 51.3 km)	39.6 (75th% = 67.6 km)	34.5 km	**0.002494**	**0.002494**	**0.002494**	**0.002494**
	DP15	2 (75th% = 5)	0 (75th% = 1)	1	**3.015e–11**	**3.015e–11**	**3.015e–11**	—
	WB abundance	3 (75th% = 4.2)	3 (75th% = 5.5)	3	0.5907	0.5907	0.5907	0.5907
	Suitability	0.23 (75th% = 0.4)	0.23 (75th% = 0.3)	0.23	0.6624	0.6624	0.6624	0.6624
	Forest coverage	0 (75th% = 0)	0 (75th% = 0)	0	0.2175	0.2175	0.2175	0.2175
	Water in 1 km	0 (75th% = 0)	0 (75th% = 0)	0	**0.009208**	**0.009208**	**0.009208**	**0.009208**

Potential transmission of ASF by arthropod vectors	Insect nets everywhere	19	43	62	0.5425	0.5425	0.5425	0.5425
	Insecticide	22	54	76	**0.1319**	**0.1319**	**0.1319**	**0.1319**
	Median of total number of midges on each farm	30	18	20 (range: 0–370)	0.3689	0.3689	0.3689	0.3689
	Prop of positive midges pools	7	1	8	**0.001929**	**0.001929**	**0.001929**	**0.001929**
	Median of total number of *S. calcitrans* on each farm	0	2	1.5 (range: 0–123)	**0.1497**	**0.1497**	**0.1497**	**0.1497**
	Prop. positive *S. calcitrans* pools	2	0	2	**0.1111**	**0.1111**	**0.1111**	**0.1111**

*Note:* NA indicates not applicable. — indicates no results were obtained for this variable in the univariate model. Near.DP: Distance (m) to nearest domestic pig outbreak; Near.WB, Distance (m) to nearest wild boar outbreak.

^a^Complete dataset: Four entire variables with some ambiguous responses were removed (that is, “Outdoor activities,” “Unusual events,” “Contact with case farms,” and “Manure from other holdings.”

^b^Ambiguous responses were imputed using a Random Forest model.

^c^Ambiguous responses were imputed as positive.

^d^Ambiguous responses were imputed as negative.

*⁣* ^*∗*^Full description of the variables is available in Supporting Information S1.

**Table 2:** Significant variables for ASF incursion by use of a conditional logistic regression model and a “Complete” dataset (i.e., variables with missing values or “I don't know” answers from some farms were excluded from the dataset), based on 110 farms located in Romania, Lithuania, and Poland.

Variable	OR	CI (2.5%−97.5%)	Se	*p*-Value
Attractive crops	8.55991	1.78–41.18	0.80143	0.007383
Pigs introduced in HRP	0.17947	0.03 – 1.07	0.91167	0.059541
Carcasses collection	0.07377	0.01 – 0.51	0.99118	0.008539
Minimum distance from DP outbreaks	0.04315	0.007 – 0.27	0.93084	0.000734

**Table 3:** Conditional logistic regression in “imputed with random forest” dataset.

Variable	OR	CI (2.5%−97.5%)	Se	*p*-Value
Bedding	8.65298	1.34845–55.5261	0.94846	0.02290
Manure from other holdings	6.72219	1.33560–33.8333	0.82452	0.02084
Insect nets everywhere	0.22371	0.05009–0.9991	0.76352	0.04986
Minimum distance from DP outbreaks	0.09627	0.02315–0.4004	0.72722	0.00129

**Table 4:** Conditional logistic regression in “Positive” dataset.

Variable	OR	CI (2.5%−97.5%)	Se	*p*-Value
Attractive crops	9.82029	1.6–60.2	0.92476	0.013499
Nonprofessional visitors	0.34842	0.14–0.85	0.45775	0.021259
Manure from other holdings	21.29411	2.01–225.56	1.20417	0.01109
Carcasses collection	0.02976	0.002–0.56	1.49375	0.018634
Minimum distance from DP outbreaks	0.03270	0.005–0.24	1.01746	0.000775
Number of biting midges	3.62788	1.1–12–1	0.61399	0.035834

**Table 5:** Conditional logistic regression in “Negative” dataset.

Variable	OR	CI (2.5%−97.5%)	Se	*p*-Value
Attractive crops	8.5876	1.6871235–43.7116	0.8303	0.0096
Unusual event	107.2683	4.7137895–2441.02	1.5943	0.00336
Fenced holding	13.5248	0.79221–230.8975	1.4477	0.07201
Carcasses storage	0.1439	0.02304356–0.8983	0.9345	0.03801
Shared machinery	9.4822	0.909922–98.81212	1.1958	0.05997
Number of stomoxys	0.1593	0.03775384–0.67206	0.7345	0.01238

**Table 6:** Covariates.

Variable name	Explanation	Category
Total.pigs	Total number of pigs in the holding	Continuous

On_farm_slaughter	1 = pigs slaughtered in the holding 0 = pigs not slaughtered in the holding	Categorical-binary

Other.animals	1 = there are other animals in the holding 0 = no other animals are kept in the holding	Categorical-binary

Attractive.crops	1 = there are any attractive crops for wild boar within 100 m to the holding 0 = no attractive feed source for wild boar near the holding	Categorical-binary

Grass_hay	1 = hay or grass is part of the type of feed given 0 = no hay or grass given	Categorical-binary

Tap_only	1 = tap water is provided to the animals as drinking water 0 = other type of drinking water provided	Categorical-binary

Disinfection	1 = there is a point of disinfection for the wheels at the entrance of the holding 0 = no pint of wheels disinfection	Categorical-binary

Professionals.visits.HRP	Number of professional visits occurred on average on a daily basis within the 6 weeks before confirmation of ASF	Continuous

Nonprof.visitors.HRP	Number of people entering the holding for nonprofessional reasons on average on a daily basis within the 6 weeks before confirmation of ASF	Continuous

Unusual.event	1 = unusual event occurred in the 6 weeks before the confirmation of the outbreak 0 = no unusual event occurred	Categorical-binary

Bedding	1 = any type of bedding is used 0 = no bedding at all is used	Categorical-binary

Fenced.holding	1 = the holding is fenced to avoid contact with wild animals 0 = the holding is not fenced	Categorical-binary

Carcasse.storage	1 = there is a closed carcasses storage 0 = no closed carcasses storage in the holding	Categorical-binary

Manure.from.other.holdings	1 = manure from other holding is spread on neighboring farmlands situated directly next to the stable (<500 m) 0 = no manure from other holding is spread on the neighboring farmlands	Categorical-binary

Pigs.introduced.in.HRP	1 = new pigs/piglets were introduced in the establishment within 6 weeks before confirmation of ASF 0 = no new pigs introduced in the HRP	Categorical-binary

Contact.case.farms	1 = there were any possible contact with case farms within the 6 weeks before confirmation of ASF 0 = no contact with case farms within the HRP	Categorical-binary

Outdoor	1 = workers of the holding carry out any outdoor activity in wild boar habitat 0 = none of the worker carry out outdoor activity in wild boar habitat	Categorical-binary

Workers.food	1 = workers are allowed to bring their own food in the holding 0 = workers are not allowed to bring their own food	Categorical-binary

Carcasses.collection	1 = carcasses can be collected by the rendering company without entering the holding 0 = carcasses cannot be collected by the rendering company from the public road	Categorical-binary

Feed.deliver	1 = feed can be delivered from the public road without entering the premises 0 = feed cannot be delivered from the public road	Categorical-binary

InsectNestseverywhere	1 = there are intact insect nests placed in front of every air intake and window 0 = no insect nests placed or not on every air intake/window	Categorical-binary

Insecticide	1 = they apply insecticide in or around the pig shed 0 = no insecticide used	Categorical-binary

All.in.all.out	1 = all-in all-out procedure applied 0 = not applied	Categorical-binary

Shared.machinery	1 = crop machinery shared with other pig farmers 0 = crop machinery not shared	Categorical-binary

Near.DP	Distance (m) to nearest domestic pig outbreak	Continuous

Near.WB	Distance (m) to nearest wild boar outbreak	Continuous

DP1	Number of domestic pig outbreaks within a distance of 1 km	Continuous

DP5	Number of domestic pig outbreaks within a distance of 5 km	Continuous

DP10	Number of domestic pig outbreaks within a distance of 10 km	Continuous

DP15	Number of domestic pig outbreaks within a distance of 15 km	Continuous

WB1	Number of wild boar outbreaks within a distance of 1 km	Continuous

WB5	Number of wild boar outbreaks within a distance of 5 km	Continuous

WB10	Number of wild boar outbreaks within a distance of 10 km	Continuous

WB15	Number of domestic pig outbreaks within a distance of 15 km	Continuous

WBabundance	Wild boar abundance around the farm (in the 2x2 square km in which the farm is located)	Continuous

Suitability	Value of the suitability of the territory for wild boar populations	Continuous

Forest.coverage	Percentage of forest coverage in the 2x2 square km in which the farm is located	Continuous

Water.in.1.km	Percentage of water in the 2x2 square km in which the farm is located	Continuous

AVG.midges	Number of biting midges sampled in the farm	Continuous

Prop.pos.pools.midges	1 = at least one pool of biting midges is positive for ASFv 0 = no positive pools detected	Categorical-binary

AVG.stomoxys	Number of *S. calcitrans* flies sampled in the farm	Continuous

Prop.pos.pools.stomoxys	1 = at least one pool of *S. calcitrans* flies is positive for ASFv 0 = no positive pools detected	Categorical-binary

**Table 7:** Results of VIF analysis.

Variable	VIF value for complete	VIF value for positive	VIF value for negative	VIF value for imputed (random forest)
Total.pigs	1.754695	1.798681	1.846133	1.827275
On_farm_slaughter	2.1960762	2.543061	2.353360	2.420901
Other.animals	4.054849	4.227759	5.196119	5.071568
Attractive.crops	2.329156	2.364077	2.483903	2.460839
Grass_hay	2.660153	2.842015	2.871223	2.779184
Tap_only	4.528693	4.808677	5.285579	5.289538
Disinfection	3.967279	4.491642	3.925207	3.938294
Professionals.visits.HRP	1.699330	2.148713	1.946166	1.899185
Nonprof.visitors.HRP	1.985833	2.445328	2.677619	2.707976
Unusual.event	—	3.488576	3.208338	3.213022
Bedding	2.687116	2.762167	3.075249	3.062062
Fenced.holding	3.115874	3.575165	3.331923	3.383485
Carcasse.storage	3.171010	3.298037	3.230112	3.201320
Manure.from.other.holdings	—	1.899601	2.216030	2.597497
Pigs.introduced.in.HRP	1.699841	1.835346	1.841628	1.844080
Contact.case.farms	—	2.952174	2.273472	2.253781
Outdoor	—	2.089055	1.775358	1.766174
Workers.food	2.364477	2.319483	2.279192	2.237949
Carcasses.collection	2.491105	2.615313	2.716765	2.697275
Feed.deliver	1.831732	2.044942	1.926781	1.915016
InsectNestseverywhere	2.175431	2.244967	2.521232	2.445397
Insecticide	2.894025	3.791038	3.205460	3.150358
All.in.all.out	2.44715	2.579779	2.849790	2.841076
Shared.machinery	2.884972	2.967304	2.585007	2.711936
Near.DP	3.041761	3.743908	3.500001	3.116193
Near.WB	2.914931	3.014766	3.258223	3.276530
DP1	7.763732	10.508812	8.085516	7.862618
DP5	8.450186	8.686217	7.904997	7.891564
DP10	4.691054	5.661208	6.254776	6.130506
DP15	3.48015	4.386051	5.059897	4.981168
WB5	6.506595	7.176016	7.341762	6.834231
WB10	17.992615	20.910184	18.995849	19.086529
WB15	10.22787	13.409434	10.908901	11.571960
Wbabundance	2.741821	2.956491	2.998937	3.043373
Suitability	1.847609	1.869231	1.990743	1.972053
Forest.coverage	1.981832	1.955801	1.972622	1.944553
Water.in.1.km	2.390066	2.714543	2.642560	2.558996
AVG.midges	2.271915	2.362755	2.346682	2.424221
Prop.pos.pools.midges	3.118224	3.154859	3.400272	3.474181
AVG.stomoxys	1.975471	1.973549	2.069656	2.045412
Prop.pos.pools.stomoxys	1.552175	1.917850	1.618967	1.648038

## Data Availability

The raw data on vector surveillance conducted at the outbreak and case farms are available via GBIF at https://doi.org/10.15468/rm3g5q. Other farm-level data are not publicly available due to personal data protection considerations.

## Supporting Information

Supporting Information Suppl_ 1_Questionnaire: Questionnaire performed on the farms that participated in the studies.
