# Supplementary material for: Prospective Case–Control Study of Determinants for African Swine Fever Introduction in Commercial Pig Farms in Poland, Romania, and Lithuania
Source: Transbound Emerg Dis. 2025 May 22;2025:5419764. doi: 10.1155/tbed/5419764 (PMC12122119; doi:10.1155/tbed/5419764)
Supplement: Supporting Information — Suppl_ 1_Questionnaire: Questionnaire performed on the farms that participated in the studies. [file 5419764.f1.pdf]

Questionnaire on ASF in domestic pig farms in Romania/Lithuania/Poland

Fields marked with * are mandatory.

OBJECTIVES

The purpose of this survey is to collect epidemiological data as well as any available information on the development of ASF in Romania/Poland/Lithuania to:

- Perform an analysis of the temporal and spatial patterns of ASF in domestic pigs, and
- Analyse the risk factors involved in the occurrence of ASF virus in **commercial pig farms**

INSTRUCTIONS

Please, carefully **read the instructions** before completing the questionnaire.

- A commercial pig farm is a holding where pigs are bred for commercial purposes. For this study, **only commercial pig farms of 30 or more pigs will be included**.
- Case (outbreak) farms will be farms where ASF has been confirmed.
- A list of 10 randomly selected control farms from the same county as case farm will be provided, matching on size (e.g. 30-200; 201-1000; >1000) each case farm. If a holding is selected as control holding, but preventive culling has been applied, or the control holding is suspected or has become (in the meanwhile) positive, please go to the next control holding on the list provided.
- High Risk Period (HRP) is defined as the length of time between possible introduction date and detection of the disease, during which no control measures have yet been implemented and disease continues to spread. To harmonize answers, we have arbitrarily defined a time lapse of 6 weeks.
- Note that you will be asked to provide the GPS location of the holding. Please, be equipped with a device that can retrieve this information (e.g. smartphone), or, if not possible to use a device, complete this information before the visit.

FIRST PART: TO BE FILLED IN **BEFORE THE VISIT** of the HOLDING by the team member appointed to perform the survey

* **1. Please, select your country:**

- Lithuania
- Poland
- Romania

* **2. Full name of team member appointed to carry out the questionnaire:**

* **3. Email address:**

Information about the holding

* **4. Holding ID:**

* **5. Disease status of pig herd:**

- Outbreak
- Control

SECOND PART: to be filled in DURING the visit

11. GPS coordinates (WGS84 coordinate system):

Example: Barcelona (Spain) has coordinates 41.403380 latitude, 2.174030 longitude

* Latitude

* Longitude

* **12. Date of the visit**

Holding animals' information

* **13. Kindly provide the total number of pigs in the holding (all animals including sucklers):**

* **14. Please, specify the number of each kind of pigs in the holding:**

Small sucklers in farrowing boxes/pens (please provide an estimate if the exact number is not known):

* Weaned piglets (7-30 kg = piglets before they go to fattening)

* Breeding sows:

* Breeding boars:

* Fattening male pigs:

* Fattening female pigs:

* Fattening pigs (when sex is unknown):

* **15. Where are the pigs slaughtered?**

- ○ On the holding (including own slaughterhouse)
- ○ In a contracted slaughterhouse
- ○ No slaughter on farm (for example if they are all sold)

* 16. Does the farm owner have other pig farms?

- ○ Yes
- ○ No

* 17. Are there **other animal species bred/kept IN** the **pig holding**?

More than one answer is possible

- ☐ Bovine
- ☐ Ovine
- ☐ Caprine
- ☐ Poultry
- ☐ Horses
- ☐ Pets (dogs, cats)
- ☐ Rabbits
- ☐ Other
- ☐ No other species

* 18. Are there **other animal species bred/kept OUTSIDE** the **pig holding**?

More than one answer is possible

- [ ] Bovine
- [ ] Ovine
- [ ] Caprine
- [ ] Poultry
- [ ] Horses
- [ ] Pets (dogs, cats)
- [ ] Rabbits
- [ ] Other
- [ ] No other species

Wild boar information

*** 19. Have you, or somebody else, ever seen a wild boar or other pigs roaming near (i.e. within 100 m) your farm?**

- ○ Yes
- ○ No

*** 20. Did you ever notice crossbred pigs born in your holding?**

- ○ Yes
- ○ No

*** 21. Have you, or somebody else, ever seen a wild boar body / body remains in the vicinity of your farm?**

- ○ Yes
- ○ No

*** 22. Could a wild boar access the feed storage?**

- ○ Yes
- ○ No

*** 23. Could a wild boar access the bedding storage?**

- ○ Yes
- ○ No

*** 24. Are there any attractive feed sources for wild boar (e.g. crops or trees) around/near (i.e. within 100 m) the holding?**

- ○ Yes
- ○ No

*** 25. Is there any wild boar baiting or feeding site in the surroundings (i.e. within 100 m) of the farm (even if forbidden)?**

- ○ Yes
- ○ No

Feed and drinking water

*** 26. What type of feed is given on your holding?**

More than one answer is possible

- [ ] Industrial compound feed: mesh
- [ ] Industrial compound feed: pellets
- [ ] Wet feed (for example buttermilk, whey)
- [ ] Cereals (including cereals used in your own on-farm milling and mixtures)
- [ ] Hay
- [ ] Fresh grass
- [ ] On-farm milling and mixture

*** 27. Are there any signs of use of kitchen waste (to be answered by official vet)?**

- ( ) Yes
- ( ) No

*** 28. Do you feed kitchen waste (to be answered by farmer)?**

- ( ) Yes
- ( ) No

*** 29. What kind of drinking water is provided to the animals?**

More than one answer is possible

- [ ] Fountain water (pumped from groundwater)
- [ ] Rain water stored in holding's own reservoir (e.g. tank, container, basin...)
- [ ] River/lake water
- [ ] Tap water (drinking / cleaning / disinfected water)

Biosecurity

*** 30. Did pigs have access to any type of outdoor areas on your holding within the last 6 weeks before the confirmation of ASF on the case farm (or the corresponding case farm in case the interview is on a control farm)?**

- ( ) Yes
- ( ) No

*** 31. Did you introduce agriculture machinery and/or equipment potentially in contact with pigs within the 6 weeks before confirmation of ASF on the case farm (or the corresponding case farm in case the interview is on a control farm)?**

More than one answer is possible

- [ ] Yes, purchased new from dealer
- [ ] Yes, second hand or borrowed
- [ ] No

*** 33. Is there a point of disinfection for the wheels at the entrance of the holding?**

- ( ) Yes
- ( ) No

*** 34. How many visits of professionals on the holding (number of people entering the farm for professional reasons) occurred on average on a daily basis within the 6 weeks before confirmation of ASF on the case farm (or the corresponding case farm in case the interview is on a control farm)?**

Private veterinarians:

* Official veterinarians:

* Farm workers:

* Consultants (industry reps…):

* Suppliers of farm utensils/feed:

* Live animal traders:

* Others:

* If OTHERS, please specify which:

*** 35. How many visits of professionals in the pig shed (number of people entering the farm for professional reasons) occurred on average on a daily basis within the 6 weeks before confirmation of ASF on the case farm (or the corresponding case farm in case the interview is on a control farm)?**

Private veterinarians:

* Official veterinarians:

* Farm workers:

* Consultants (industry reps…):

* Suppliers of farm utensils/feed:

* Live animal traders:

* Others:

* If OTHERS, please specify which:

* **36. How many visitors (number of people belonging to family, friends, students, entering the farm for personal reasons) entered the holding on average on a daily basis within the 6 weeks before confirmation of ASF on the case farm (or the corresponding case farm in case the interview is on a control farm)?**

visitors

* **37. Was there any unusual event within the 6 weeks before the confirmation of the outbreak on the case farm/on the control farm corresponding to the case farm in case the interview is on a control farm?**

More than one answer is possible

- ☐ Construction work new building
- ☐ Change of staff
- ☐ Family/social event
- ☐ Break in biosecurity routine
- ☐ Other
- ☐ No
- ☐ I don't know

* **38. What kind of bedding is used in the sheds?**

More than one answer is possible

- ☐ Straw
- ☐ Wood chips

- [ ] Sawdust
- [ ] None
- [ ] Other

* **39. Is the holding fenced to avoid contact with wild animals (e.g. wild boar, wolves, foxes, jackals,...)?**

- ○ Yes
- ○ No

* **40. Is there a closed carcass storage (e.g. container, cooling facility, premise)?**

- ○ Yes
- ○ No

* **41. Is manure from other holdings spread on neighbouring farmlands situated directly next to your stables (< 500 meters)?**

- ○ Yes
- ○ No
- ○ I don't know

* **42. Were NEW pigs and/or piglets introduced or purchased in the establishment within the 6 weeks before confirmation of ASF on the case farm?**

- ○ Yes
- ○ No

* **43. Were pigs sold from the establishment within the 6 weeks before confirmation of ASF on the case farm (or the corresponding case farm in case the interview is on a control farm)?**

- ○ Yes
- ○ No

* **44. Were there any possible contacts with case farms within the 6 weeks before confirmation of ASF on the case farm? (e.g. natural mount, artificial insemination, shared material/objects/vehicles, external visitors, etc.)**

- ○ Yes
- ○ No
- ○ I am not sure/I do not know

* **45. Does anyone among the workers of your holding carry out any outdoor activity (in wild boar habitat)?**

More than one answer is possible

- [ ] Work (forest management, agricultural activities, bee-keeping, ...)
- [ ] Leisure (hiking, mountain climbing, mountain biking, bird watching,...)
- [ ] Hunting
- [ ] None of the above
- [ ] I don't know
- [ ] Other

* **46 . Are the workers allowed to bring their own food in the holding?**

- Yes
- No

* **47. Does anyone among the workers of your holding have contact to pigs on other farms or in their private life (including other premises owned by you)?**

- Yes
- No

* **48. Can carcasses be collected by the rendering company from the public road, without entering to the premises?**

- Yes
- No

* **49. Can the feed be delivered from the public road without entering the premises?**

- Yes
- No

* **50. How are pigs loaded, when they leave for selling or slaughter?**

- Truck enters farm and pigs can possibly return to shed from the truck
- Truck enters farm and pigs cannot return to shed from the truck
- Truck does not enter farm and pigs cannot return to shed

* **51. Are pig pens accessible to visitors and workers only through the locker room?**

- Yes
- No

* **52. Is there a rodent control management plan implemented?**

- Yes
- No

* **53. Are there intact insect nets placed in front of the air intakes and the windows in the sheds?**

- Yes, on all air intakes and the windows
- Yes, but not on all air intakes and the windows
- No

* **54. Do you apply insecticide in or around your pig shed?**

- Yes
- No

* **55. What product is used for disinfection of baths/ boot washers/stables at the holding?**

Name of commercial product

* **56. Is a quarantine period respected if new pigs arrive or are purchased in your holding?**

- Yes
- No

* **57. Is the procedure all-in/all-out for all pigs on farm implemented?**

- Yes
- No

Seasonality

* **58. Do you share any crop harvesting machinery with other pig farmers?**

- Yes
- No

* **59. Is there a change in diet of the pigs over summer?**

- Yes
- No

Open question for case farms

* **60. Is there any other potential risk factor that we did not inquire about, that you consider the possible reason of introduction of the virus on your farm?**

Background Documents

Data Protection Note [ENG]

Data Protection Note [LT]

Data Protection Note [PL]

Data Protection Note [RO]
